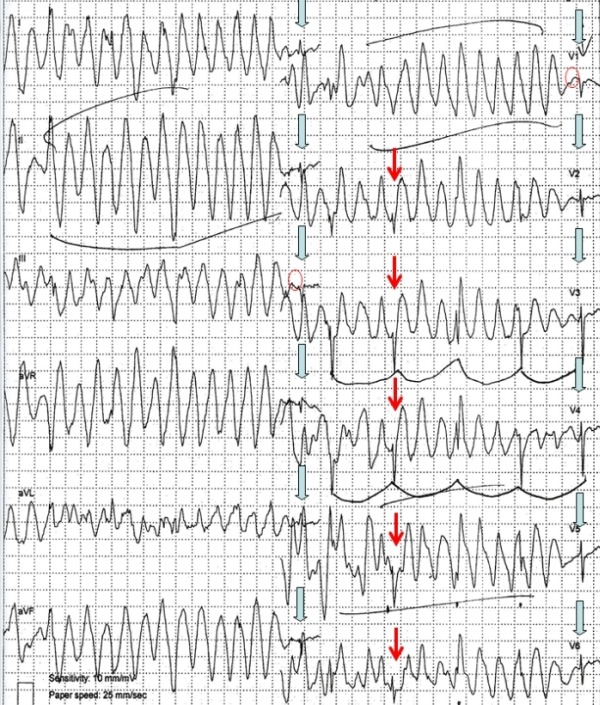# Pseudo-polymorphic Ventricular Tachycardia in a 12-lead Holter Recording

**DOI:** 10.1016/s0972-6292(16)30632-5

**Published:** 2013-06-25

**Authors:** George Nikitas, Christos Maniotis, George Manolis, Athanasios G Manolis

**Affiliations:** Hellenic Red Cross Hospital of Athens, Greece

**Keywords:** Holter recording, artifacts, polymorphic ventricular tachyarrhythmia

## Abstract

We present an image of pseudo-polymorphic ventricular tachycardia recording on a 12-lead surface ECG Holter. Although at first glance the appearance of the recording resembled polymorphic ventricular tachycardia, careful investigation revealed normal electrocardiographic findings.

A 58 year old asymptomatic hypertensive patient was referred to our center for further investigation (performance of an electrophysiological study) due to a polymorphic ventricular tachycardia (VT) seen on the 12-lead surface ECG Holter strip recording (Figure 1). The patient was a professional truck driver and the 24-hour ECG Holter recording had been performed for insurance reasons [1]. After a careful revision of the tracing, it was noted that the last QRS complexes in both the limb and the precordial leads (blue arrows) were narrow and exhibited near normal morphology. Similar complexes also appeared during the ongoing tachycardia between the wide QRS complexes (red arrows). The underlying rhythm was of sinus origin (circle), as assumed by a P wave preceding the last QRS complex in the majority of the leads. This was confirmed by the appearance of regular QRS complexes with normal cycle length among the "polymorphic tachycardia" waveforms in the precordial lead recordings. The diagnosis of polymorphic VT was based on the wide QRS complexes, which turned out to be electrocardiographic artifacts. Indeed, an accurate analysis of the trace revealed an underlying normal heart rhythm. The mechanism behind this artifact lies in the "tapping" of the external electrodes depending on the patient's activities during the recording. Common sources of artifacts in analog recorders include electrode displacement, cable rupture, myopotentials, body movement or repeated regular tapping on the chest wall during chest physiotherapy or breathing exercises [2]. Based on the aforementioned analysis of the Holter tracing, we decided not to conduct any further diagnostic testing. Presently, two years after this incident, the patient is still asymptomatic and in good physical condition. Electrocardiographic artifacts can closely simulate VT and can sometimes be misdiagnosed as such, resulting in unnecessary and potentially dangerous medical treatment or interventions [3]. In order to prevent artifacts during a Holter recording, the integrity of the electrodes must be evaluated and the patient be advised to avoid intense body movements or exercise. In addition, physicians must be experienced and able to recognize such artifacts [4] to avoid an erroneous diagnosis.

## Figures and Tables

**Figure 1:**